# Optimum duration of breast cancer follow-up: a continuing controversy

**DOI:** 10.1038/sj.bjc.6603979

**Published:** 2007-09-11

**Authors:** L Maraqa, M Lansdown

**Affiliations:** 1 Breast Unit, Leeds General Infirmary, Great George Street, Leeds LS1 3EX, UK

**Sir**,

The recent article by Montgomery *et al* (2007) has raised yet again the controversies regarding optimum duration and method of breast cancer follow-up. In fact, there has been little enthusiasm in adopting the 2002 National Institute for Clinical Excellence guidelines. The authors correctly questioned the basic assumptions behind these guidelines but also agreed that clinical reviews were ineffective in their analysis. It was also stated that clinically detected cancers had worse outcomes. With regards to this particular point, we propose an alternative analysis and interpretation of the data presented. We feel without this clarification, some patients may be discharged on such an assumption.

Figures 4 and 5 of the above mentioned article included only 48 out of 110 regional relapses, yet the authors did not explain why isolated contralateral recurrences were excluded. Additionally, when comparing these 48 cases with Table 3 of the same article, it was evident that ipsilateral axillary recurrences were also excluded without justification. This is statistically incorrect as only 4 out of the 25 pure ipsilateral axillary recurrences were evident on mammography, thus obscuring any potential benefit of clinical examination.

To re-analyse the published data, we reproduced an approximation of Figure 4 using information contained in the original analysis, see Figure 1:

First, we question the validity of any statistics proposed on small subsets – note only four cases were included in the ‘clinically detected’ group. Perhaps, a more relevant analysis would be to group all palpable cancers since these are equally ‘clinically’ detectable. By performing this analysis, the statistical significance between mammography and palpable tumours is lost (log rank *P*=0.135), Figure 2, with no difference in outcome. We were not able to reproduce a full analysis using lymph node detection as follow-up times were not included in the paper.

Second, the authors did not specify their definition of survival, that is, breast specific or overall survival, thus compounding the difficulties in data interpretation. This is particularly relevant in small groups and represents a strong confounding factor.

We appreciate the need to improve clinic efficiency but the evidence presented in this paper does not lead us to agree with the authors' conclusion that relapses diagnosed clinically are in general associated with poorer outcomes and does not take the argument against clinical follow-up any further.

## Figures and Tables

**Figure 1:**
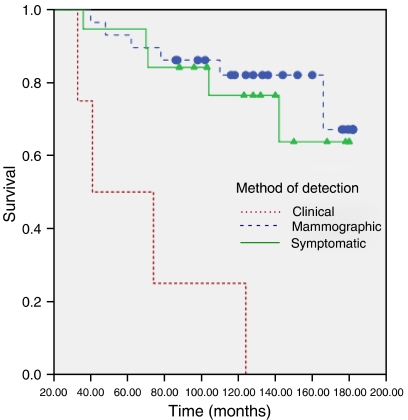
Reproduced survival curves using information from the original paper. Curves are approximates for patients with ipsilateral breast relapse. Patients with pure axillary disease were excluded.

**Figure 2:**
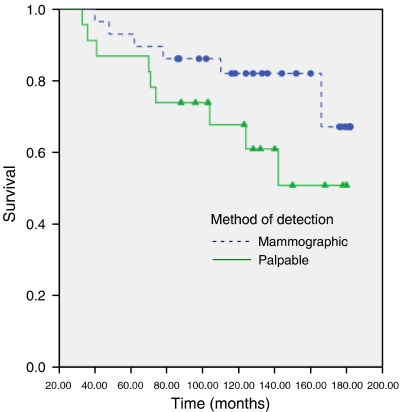
Our interpretation of Figure 1 after reclassifying groups into palpable *vs* mammographically detected cancers.
